# Specific cyprinid HIF isoforms contribute to cellular mitochondrial regulation

**DOI:** 10.1038/s41598-020-74210-w

**Published:** 2020-10-14

**Authors:** Jing Chen, Lihong Guan, Ming Zou, Shunping He, Dapeng Li, Wei Chi

**Affiliations:** 1 College of Fisheries, National Demonstration Center for Experimental Aquaculture Education, Huazhong Agricultural University, Wuhan, 430070 China; 2 College of Life Science and Technology, Xinxiang Medical University, Xinxiang, China; 3 Institute of Hydrobiology, Chinese Academy of Sciences, Wuhan, China; 4 Hubei Provincial Engineering Laboratory for Pond Aquaculture, Wuhan, China

**Keywords:** Cell biology, Evolution, Molecular biology, Physiology

## Abstract

Hypoxia-inducible factor 1 (HIF-1) functions as a master regulator of the cellular response to hypoxic stress. Two HIF-1α paralogs, HIF-1αA and HIF-1αB, were generated in euteleosts by the specific, third round of genome duplication, but one paralog was later lost in most families with the exception of cyprinid fish. How these duplicates function in mitochondrial regulation and whether their preservation contributes to the hypoxia tolerance demonstrated by cyprinid fish in freshwater environments is not clear. Here we demonstrated the divergent function of these two zebrafish Hif-1a paralogs through cellular approaches. The results showed that Hif-1aa played a role in tricarboxylic acid cycle by increasing the expression of *Citrate synthase* and the activity of mitochondrial complex II, and it also enhanced mitochondrial membrane potential and ROS production by reducing free Ca^2+^ in the cytosol. Hif-1ab promoted intracellular ATP content by up-regulating the activity of mitochondrial complexes I, III and IV and the expression of related genes. Furthermore, both the two zebrafish Hif-1a paralogs promoted mitochondrial mass and the expression level of mtDNA, contributing to mitochondrial biogenesis. Our study reveals the divergent functions of Hif-1aa and Hif-1ab in cellular mitochondrial regulation.

## Introduction

As one of the most important environmental factors for animal life, oxygen is the terminal electron acceptor in the process of aerobic energy production. Oxygen-sensing pathway can enhance hypoxia tolerance by inhibiting translation and activating transcription. Metazoans have evolved complicated cellular metabolism and physiology systems to maintain oxygen homeostasis, including the induction of biochemical responses at low oxygen levels^1,2^. Hypoxia-inducible factors (HIFs) are the main regulators of the cellular response to hypoxia and control the expression of target genes involved in many processes, including angiogenesis, erythropoiesis, glycolysis, glucose and iron transport^1,3^.

HIF exists in all metazoans. It consists of two subunits: HIF-α and HIF-β, which determines the transcriptional activity of HIF and known as aryl hydrocarbon nuclear translocator, respectively^1^. Under hypoxic conditions, HIF-1α dimerizes with HIF-1β subunit and forms an active transcription factor capable of activating the expression of target genes by binding to the hypoxia-responsive elements (HRE). The transcriptional activity of HIF mainly depends on the stability of the α-subunit, which is functionally inhibited and degraded by prolyl hydroxylase enzymes under normal oxygen, but stable under hypoxic condition^4^. Cobalt chloride (CoCl_2_) is a mimetic reagent used to induce hypoxia-mediated cellular responses in vitro. It has been demonstrated that CoCl_2_ stabilizes HIF-1α by inhibiting prolyl hydroxylase enzymes^5^. Invertebrates possess only one HIF-α, while jawed vertebrates that have undergone two rounds of whole genome duplication (WGD) during evolution possess three functional HIF-α isoforms: HIF-1α, HIF-2α and HIF-3α^3,6,7^. Teleosts experienced a specific third round of WGD early in their evolution^8,9^, generating two copies of each HIF-α isoform (HIF-1αA/B, HIF-2αA/B, HIF-3αA/B), then one of each A/B paralogous pair was lost in most euteleosts, except for cyprinids^10–12^.

Mitochondria are essential for cellular hypoxic response because they play a central role in energy supply, reactive oxygen species (ROS) generation and apoptosis^13^. Among the three HIF-α isoforms, HIF-1α is recognized as the most important one and has been studied extensively^14,15^. A previous study has reported that mitochondria produce a burst of ROS in response to hypoxic stress, which contribute to the stability of HIF-1^16^. Many studies support this conclusion and demonstrate that ROS produced by the mitochondrial respiratory chain is necessary for the normal induction of HIF-1^17–19^. Mitochondrial oxidative phosphorylation (OXPHOS) is the main pathway for energy production in mitochondrial glucose metabolism^20^. It can adapt to hypoxia by regulating the electron transport chain (ETC). There are four complexes in the ETC: NADH-coenzyme Q reductase (complex I), succinate-coenzyme Q reductase (complex II), ubiquinol cytochrome c reductase (complex III) and cytochrome c oxidase (complex IV). Under hypoxic condition, HIF-1α can increase the transcription level of the *PDK1* gene, which encodes a kinase that decreases the activity of pyruvate dehydrogenase and prevents its conversion to acetyl CoA, thereby attenuating mitochondrial respiratory chain, resulting in the imbalance of electron flow in the ETC and the production of a large amount of ROS^21,22^. Another adaptive response of mitochondria to hypoxia is the decrease in mitochondrial mass^23^. Qian et al. reported that hypoxic stimulation could inhibit the activity of lysine demethylase 3A and PGC-1ac, thereby reducing mitochondrial biogenesis and enabling tumor cells to adapt to hypoxic stress^24^.

Cyprinidae is the most species-rich family in freshwater fishes, comprising species that tolerate hypoxic condition and even anoxic stress better than any other vertebrate^25^. It has been reported that cyprinid HIF-α paralogs have become subfunctionalized during evolution after the third round WGD, with one of each paralogous cyprinid HIF-α pair maintaining the ancestral developmental response, while the other is more sensitive to changes in oxygen tension^11^. However, little is known about the functional divergence of the two cyprinid HIF-1α paralogs in mitochondrial regulation and whether the preservation of these two paralogs contributes to their survival in freshwater environments. The zebrafish (*Danio rerio*) belongs to the Cyprinidae family, which is established as a model organism in many studies. In the present study, we investigated how zebrafish Hif-1aa and Hif-1ab affect mitochondrial biogenesis and function, the different roles they play and whether they contribute to the hypoxia tolerance.

## Results

### Transcriptional activity of zebrafish Hif-1aa and Hif-1ab

**Figure 1:**
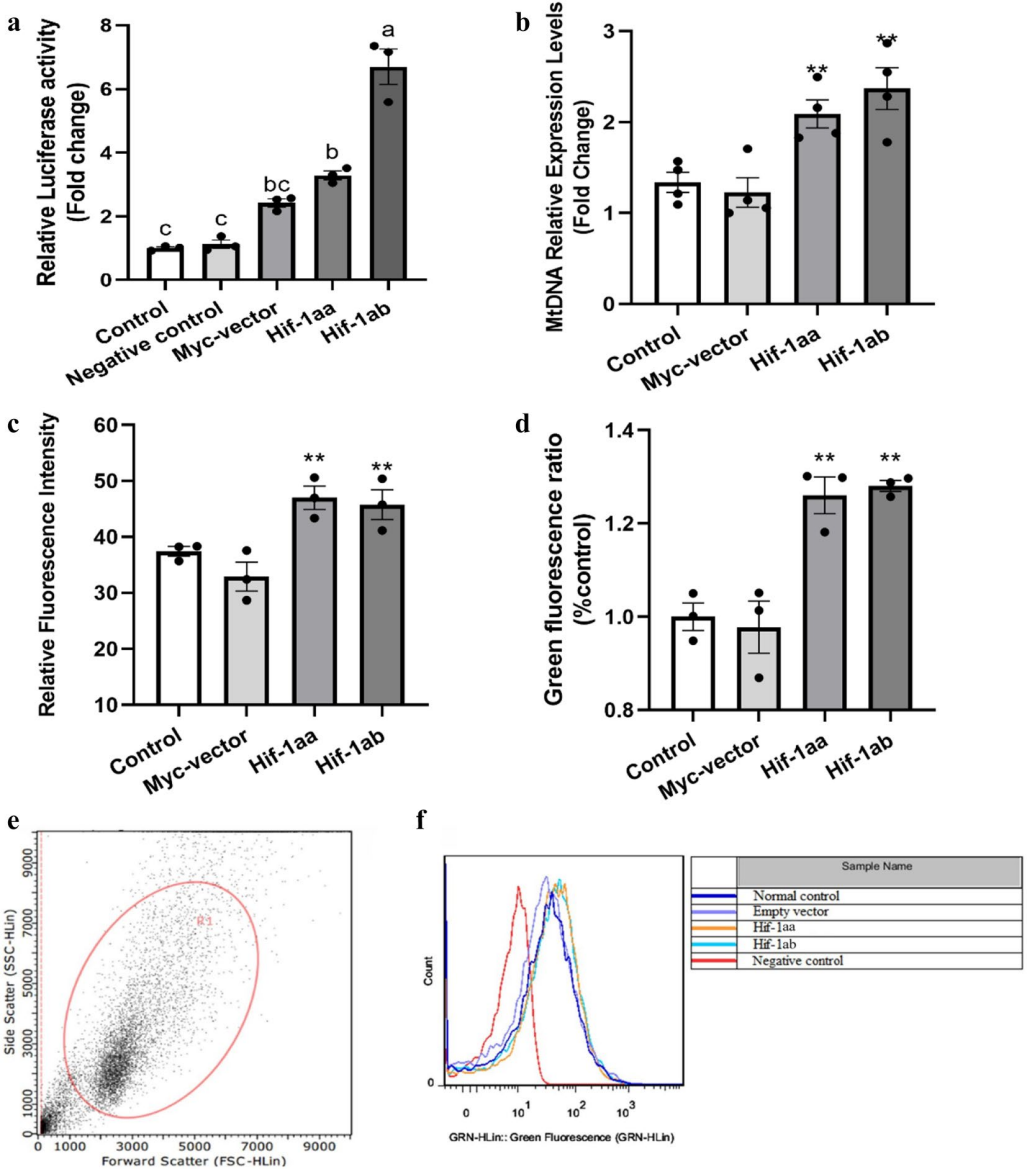
Regulation of the transcriptional activity and mitochondrial biogenesis by zebrafish Hif-1aa and Hif-1ab. (**a**) Transcriptional activity of zebrafish Hif-1aa and Hif-1ab in HEK 293 cells exposed to 100 μM CoCl_2_ for 24 h. Data are given as mean ± SE (n = 3 per group). The negative control means the empty vector co-transfected with HRE-luciferase reporter and pTK-Renilla luciferase reporter without CoCl_2_ treatment. The different letter superscripts indicate significant differences (*P* < 0.05), and the same letter indicates not significantly different (*P* > 0.05). (**b**) The expression level of mtDNA control region regulated by zebrafish Hif-1aa and Hif-1ab in HEK 293 cells exposed to 100 μM CoCl_2_ for 24 h. Data are given as mean ± SE (n = 4 per group). **p < 0.01. (**c**) Statistical analysis showed that zebrafish Hif-1aa and Hif-1ab induced an increase in relative fluorescence of intensity by flow cytometric analysis. Data are given as mean ± SE (n = 3 per group). **p < 0.01. (**d**) Statistical analysis of the green fluorescence intensity examined by the confocal Laser-scanning microscope. Data are given as mean ± SE (n = 3 per group). **p < 0.01. (**e**) Flow cytometric determination of size (forward scatter, FSC) and structure (side scatter, SSC) of living cells. Appropriate settings of FSC (logarithmic scale) and SSC (liner scale) permit detecting events scattered with a variable SSC and homogenous FSC (ellipsoid gate, R1). (**f**) Mitochondrial mass was estimated by Mito-Tracker Green staining using flow cytometry. The solid histogram represents the fluorescence of cells.

To characterize the transcriptional activity of zebrafish Hif-1aa and Hif-1ab, we co-transfected an HRE-luciferase reporter and a pTK-*Renilla* luciferase reporter together with either the indicated Myc-tagged HIF-1α construct or empty vector into HEK293 cells. The result showed that both Hif-1aa and Hif-1ab were stable and functional (*p* < 0.05, Fig. 1a), and Hif-1ab exhibited higher transcriptional activity than Hif-1aa (*p* < 0.01), suggesting that Hif-1ab was more stable. Furthermore, the empty vector group with CoCl_2_ treatment presented increased transcriptional activity compared with the negative control (without CoCl_2_ treatment) group and control group. Therefore, the result revealed that CoCl_2_ could mediated HIF stabilization in normal oxygen level.

### Function of Hif-1aa and Hif-1ab in mitochondrial biogenesis

**Figure 2:**
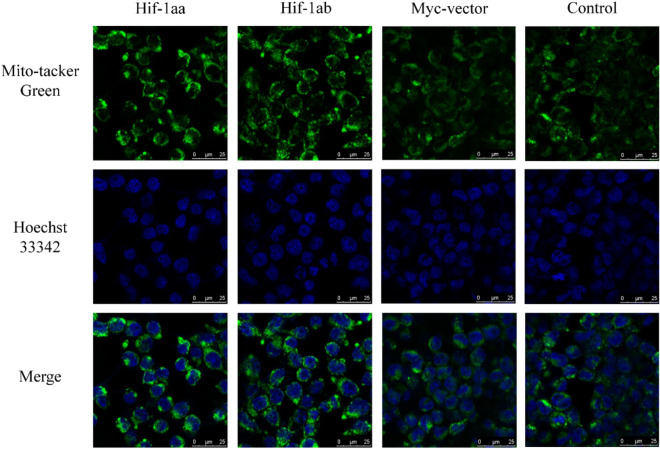
Mitochondrial mass examined by the confocal Laser-scanning microscope. Cells transfected with zebrafish Hif-1aa or Hif-1ab were stained with Mito-tracker Green to visualize the mitochondria and Hoechst 33,342 to visualize the nucleus. The higher relative fluorescence intensity represents an increase in mitochondrial mass.

To investigate whether the two Hif-1a paralogs stimulate mtDNA replication, real-time PCR was used to detect the transcription levels of the mtDNA control region (D-loop) after cells were cultured with 100 μM CoCl_2_ and incubated for 24 h, using *beta-actin* as the internal control. In cells transfected with Hif-1aa, transcription levels of the control region were significantly increased (*p* < 0.01, Fig. 1b), and a similar phenomenon was found in cells transfected with Hif-1ab (*p* < 0.01), suggesting both Hif-1a paralogs up-regulate expression of the D-loop. We further investigated the function of Hif-1aa and Hif-1ab in mitochondrial biogenesis by labelling mitochondria with a mitochondrial specific fluorescent dye (Mito-tracker Green) and quantifying mitochondrial mass by flow cytometry (Fig. 1e, f). The relative fluorescence intensity was significantly increased in cells transfected with Hif-1aa and Hif-1ab (*p* < 0.01, Fig. 1c), but no differences were observed between Hif-1aa and Hif-1ab, indicating that both paralogs promote an increase in mitochondrial mass. Furthermore, the fluorescence intensity visualized by confocal laser-scanning microscope was stronger in cells transfected with Hif-1aa and Hif-1ab (*p* < 0.01, Fig. 1d and Fig. 2). These results indicated that zebrafish Hif-1aa and Hif-1ab promoted mitochondrial biogenesis by increasing the mitochondrial mass and the transcription level of mtDNA control region.

### Effect of Hif-1aa and Hif-1ab on oxidative phosphorylation complexes

**Figure 3:**
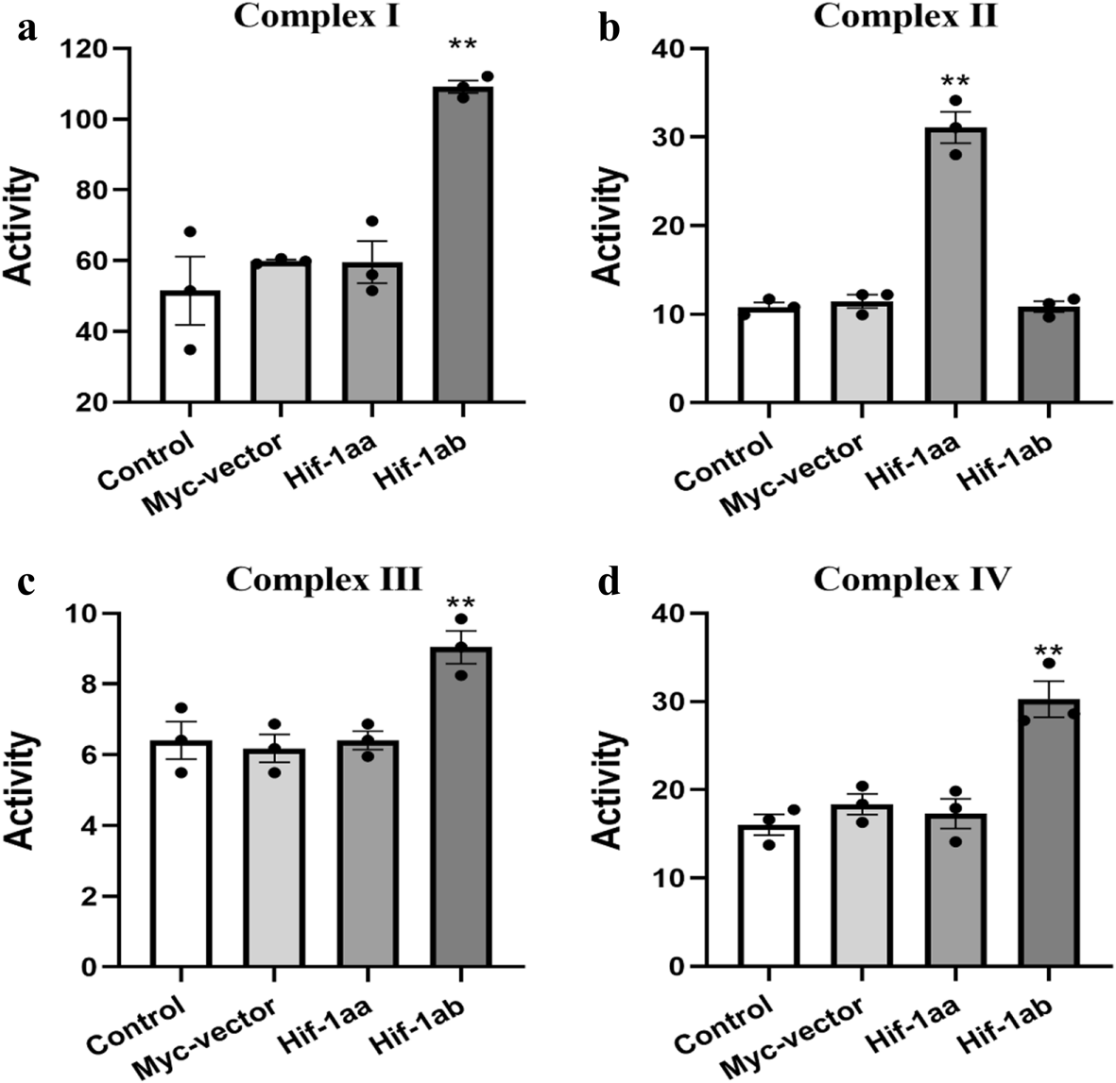
Regulation of the activities of mitochondrial OXPHOS complexes by zebrafish Hif-1aa and Hif-1ab. (**a–d**) Activities of mitochondrial complex I, II, III and IV regulated by Hif-1aa and Hif-1ab in HEK 293 cells exposed to 100 μM CoCl_2_ for 24 h. Data are given as mean ± SE (n = 3 per group). **p < 0.01.

Oxidative phosphorylation (OXPHOS) is a major metabolic pathway for ATP production. Electron transport is the most complex and productive cellular respiration pathway. The electron transport chain includes four multimeric integral membrane protein complexes, the activities of which determine mitochondrial function. To investigate the regulatory roles of Hif-1aa and Hif-1ab in oxidative phosphorylation, we tested the activities of the four mitochondrial complexes in cells transfected with zebrafish Hif-1aa or Hif-1ab. The activity of complex I was detected by using nicotinamide adenine dinucleotide (NADH) as a substrate, when it was oxidized for 1 min at 30 °C, the absorbance value was different at 340 nm and 380 nm. By calculating the change of absorbance value, the result showed that Hif-1ab significantly increased the activity of complex I compared with Hif-1aa, empty vector and control group (*p* < 0.01, Fig. 3a). The activity of complex II was detected by using 2,6-dichoroindophenol sodium (an analog of coenzyme Q) as a substrate, when it was reduced, the absorbance value changed at 600 nm over time. By calculating the change of absorbance value, we observed that Hif-1aa significantly enhanced the activity of complex II (*p* < 0.01, Fig. 3b). The activity of complex III was detected by the rate at which ubiquinol-2 reduces cytochrome c. In this study, the activity of complex III was significantly promoted by Hif-1ab (*p* < 0.01, Fig. 3c). The activity of complex IV was detected by using reduced cytochrome c as a substrate, which was converted to oxidized cytochrome c by the catalysis of complex IV, and caused changes in absorbance value at 550 nm. The result demonstrated that Hif-1ab significantly increased the activity of complex IV compared with other groups (*p* < 0.01, Fig. 3d). Therefore, these results revealed that zebrafish Hif-1aa significantly promoted the activity of mitochondrial complex II, and the activities of mitochondrial complexes I, III and IV were significantly up-regulated in cells transfected with Hif-1ab.

### Effect of Hif-1aa and Hif-1ab on mitochondrial function related genes

**Figure 4:**
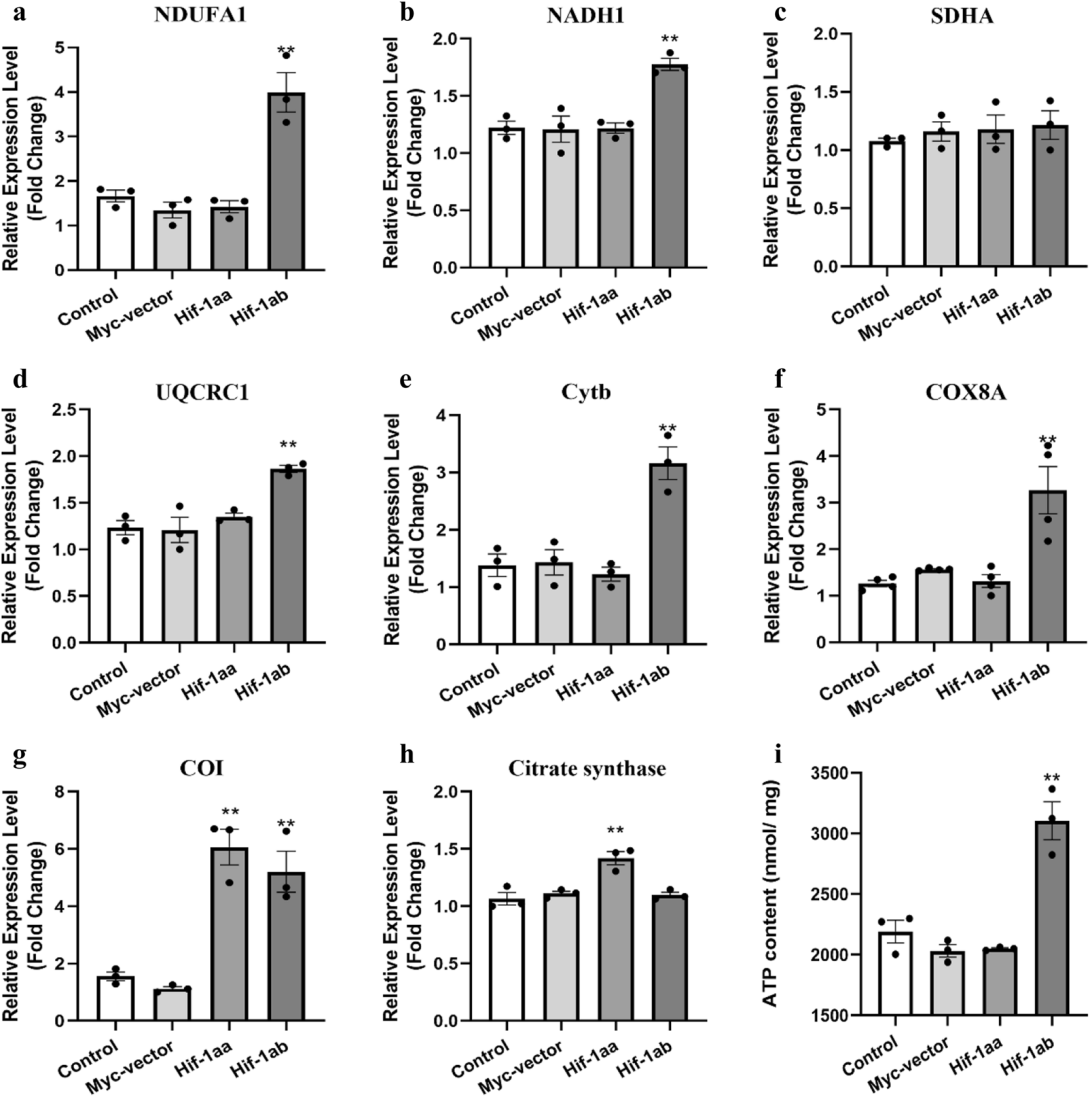
Regulation of mitochondrial function related genes and ATP production by zebrafish Hif-1aa and Hif-1ab. (**a**, **b**) The expression level of the nuclear gene (*NDUFA1*) and mitochondrial gene (*NADH1*), which related to oxidative phosphorylation complex I regulated by zebrafish Hif-1ab in HEK 293 cells exposed to 100 μM CoCl_2_ for 24 h. Data are given as mean ± SE (n = 3 per group). **p < 0.01. (**c**) The expression level of the nuclear gene (*SDHA*) related to oxidative phosphorylation complex II in HEK 293 cells exposed to 100 μM CoCl_2_ for 24 h. Data are given as mean ± SE (n = 3 per group). **p < 0.01. (**d**, **e**) The expression level of the nuclear gene (*UQCRC1*) and mitochondrial gene (*Cytb*) related to oxidative phosphorylation complex III regulated by zebrafish Hif-1ab in HEK 293 cells exposed to 100 μM CoCl_2_ for 24 h. Data are given as mean ± SE (n = 3 per group). **p < 0.01. (**f, g**) The expression level of the nuclear gene (*COX8A*) and mitochondrial gene (*COI*) related to oxidative phosphorylation complex IV regulated by zebrafish Hif-1aa and Hif-1ab in HEK 293 cells exposed to 100 μM CoCl_2_ for 24 h. Data are given as mean ± SE (n = 3 per group). **p < 0.01. (**h**) The expression level of *Citrate synthase* regulated by zebrafish Hif-1aa in HEK 293 cells exposed to 100 μM CoCl_2_ for 24 h. Data are given as mean ± SE (n = 3 per group). **p < 0.01. (**i**) ATP production regulated by Hif-1ab in HEK 293 cells exposed to 100 μM CoCl_2_ for 24 h. Data are given as mean ± SE (n = 3 per group). **p < 0.01.

To further explore the function of Hif-1aa and Hif-1ab on regulating mitochondrial complexes, we determined the transcription levels of nuclear and mitochondrial genes encoding the key enzymes in each mitochondrial complex after cells were transfected with Hif-1aa and Hif-1ab. First, we examined *NDUFA1* and *NADH1*, the nuclear and mitochondrial genes encoding essential components of complex I in the respiratory chain that transfers electrons from NADH to ubiquinone. And the transcription level of *NDUFA1* and *NADH1* were significantly up-regulated in cells transfected with Hif-1ab but not Hif-1aa (*p* < 0.01, Fig. 4a, b). We next examined *SDHA*, a gene encoding one of the four succinate-coenzyme Q reductase (complex II) subunits. The result indicated no alterations in *SDHA* expression in cells transfected with either Hif-1aa or Hif-1ab (Fig. 4c). Nuclear gene (*UQCRC1*) and mitochondrial gene (*Cytb*) play an important role in encoding one of the subunits of the respiratory chain protein ubiquinol cytochrome *c* reductase (complex III); quantitative analysis revealed significantly increased the transcriptional levels of *UQCRC1* and *Cytb* in cells transfected with Hif-1ab but not Hif-1aa (*p* < 0.01, Fig. 4d, e). For complex IV, we investigated nuclear gene *COX8A* and mitochondrial gene *COI*, which encode the essential component of complex IV. The *COX8A* expression was only significantly increased in cells transfected with Hif-1ab (*p* < 0.01, Fig. 4f), but the expression of *COI* was both up-regulated with Hif-1aa and Hif-1ab (*p* < 0.01, Fig. 4g). These results showed the correlation of nuclear and mitochondrial gene transcription in OXPHOS complexes I, III and IV, which suggested that the nuclear and mitochondrial genomic regions encoding this portion of the OXPHOS pathway are co-adapted in zebrafish Hif-1ab.

Mitochondrial complex II is a key enzyme complex in the electron transfer chain and tricarboxylic acid (TCA) cycle, which is encoded only by nuclear genome, we found that Hif-1aa promoted the activity of complex II and the expression level of *SDHA*. Citrate synthase, the first and rate-limiting enzyme of TCA cycle, catalyzes the reaction between oxaloacetate and acetyl coenzyme A to generation citrate. The results showed that Hif-1aa promoted TCA cycle by significantly increasing the expression level of *Citrate synthase* in cells (*p* < 0.01, Fig. 4h). Taken together, we consider that zebrafish Hif-1aa may play an important role in TCA.

### Hif-1ab improved intracellular ATP production

In cells, mitochondrial oxidative phosphorylation is an important source of ATP. To investigated whether the two zebrafish Hif-1a paralogs effect on intracellular ATP production, we transfected Hif-1aa and Hif-1ab into HEK293 cells (100 μM CoCl_2_, 24 h). Herein, the detection of ATP content mainly uses firefly luciferase, which can catalyse luciferin to produce fluorescence by consuming ATP. When both firefly luciferase and luciferin are in excess, the fluorescence is proportional to the ATP concentration within a certain concentration range^26^. After transfection, all samples were collected and mixed with ATP detection solution containing luciferase, and ATP content was estimated according to the strand curve. The result showed that zebrafish Hif-1ab significantly increased ATP production compared with control group and treatment groups which transfected with Hif-1aa and empty vector (*p* < 0.01, Fig. 4i), suggesting that Hif-1ab increased ATP production by enhancing the activities of complexes I, III and IV.

### Hif-1aa increased mitochondrial membrane potential

**Figure 5:**
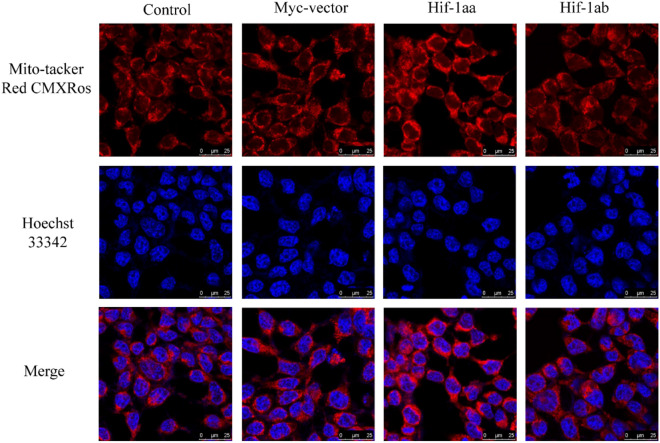
Mitochondrial membrane potentials examined by the confocal Laser-scanning microscope. Cells were stained with MitoTracker Red CMXRos to visualize the mitochondria and Hoechst 33,342 to visualize the nuclei. Higher relative fluorescence intensity represents a higher mitochondrial membrane potential.

**Figure 6:**
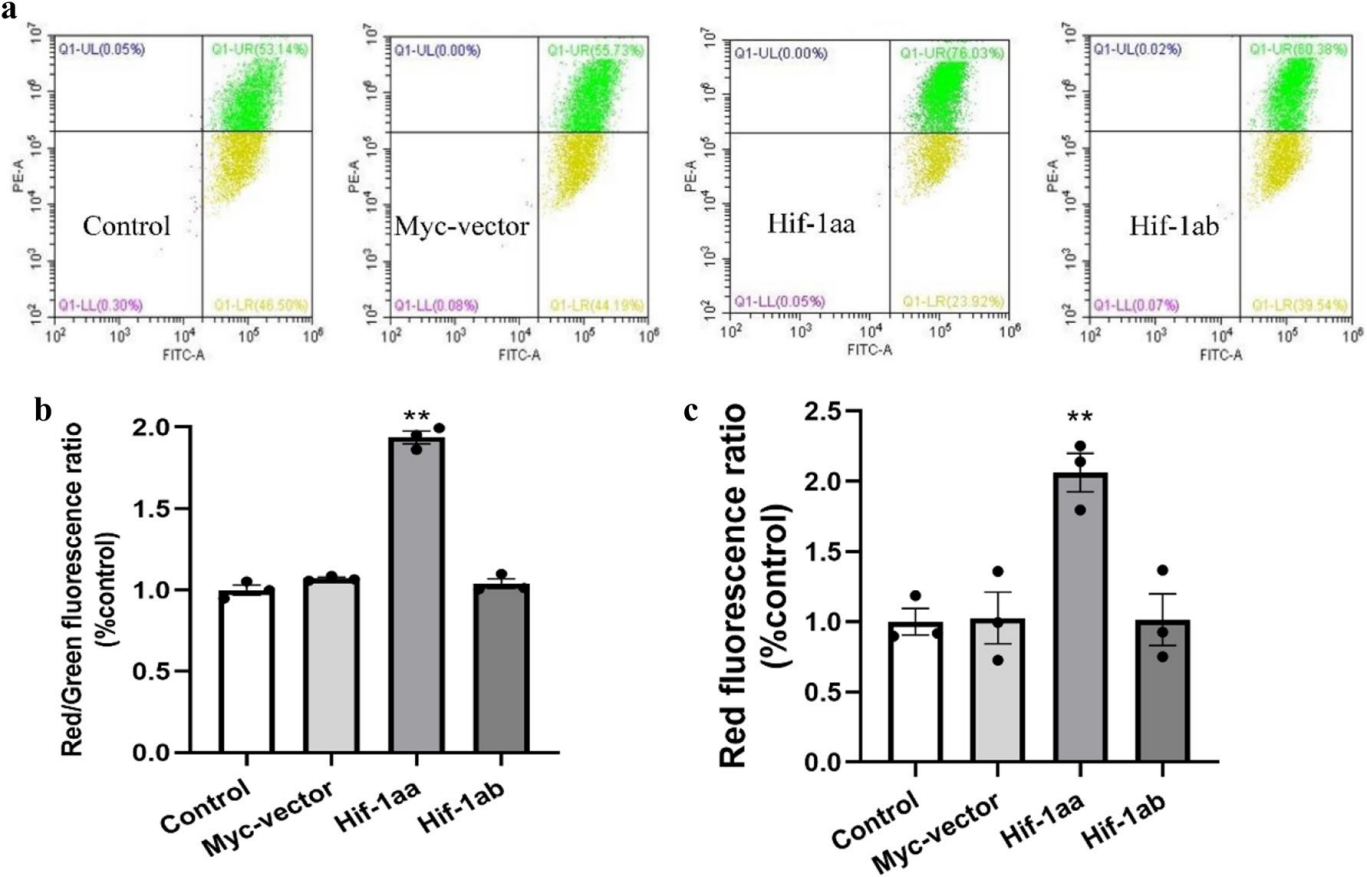
Regulation of mitochondrial membrane potentials by zebrafish HIF-1aa and HIF-1ab. (**a**) JC-1 fluorescence was showed in both the FL-1 and FL-2 channels by the flow cytometry. The data presented here indicated that the cell ratio in two channels (red and green fluorescence) were regulated by the two Hif-1a paralogs in HEK 293 cells exposed to 100 μM CoCl_2_ for 24 h. (**b**) Statistical analysis of the rate of Red/Green fluorescence intensity measured by flow cytometry. Data are given as mean ± SE (n = 3 per group). *p < 0.05. (**c**) Statistical analysis of the red fluorescence intensity examined by the confocal Laser-scanning microscope. Data are given as mean ± SE (n = 3 per group). **p < 0.01.

Considering that the membrane potential is closely related to mitochondrial function and stability, we correspondingly investigated mitochondrial membrane potential (MMP) of the cells transfected with empty vector, Hif-1aa and Hif-1ab, respectively. Unlike Mito-tracker green, which is an MMP-independent indicator of mitochondrial mass, Mito-tracker Red CMXRos is a lipophilic cationic fluorescent dye that can enter the negatively charged mitochondria, which indicates that their loading depends on ΔΨm^27^. Some other dyes, such as rhodamine 123, have proven to be very effective in MMP flow cytometric studies, but their high sensitivity to microscope lamp illumination limits the use of this dye in applications involving microscopy. However, Mito-tracker Red CMXRos can exhibit good photostability to respond to changes in MMP^28^. In this study, we observed a significant increase of fluorescence intensity in the Hif-1aa group but not in the Hif-1ab group (*p* < 0.01, Figs. 5 and 6c), suggesting an ascent of MMP with the presence of Hif-1aa. To further verify our results, the MMP was also monitored by performing JC-1 staining, which specifically measures changes in membrane potential^29^. There are two forms of JC-1: J-aggregates and monomers. The high membrane potential of cells loaded with JC-1 permits the formation of red-fluorescent J-aggregates. However, when the membrane potential is reduced, these J-aggregates dissipate into monomers, and produce a shift from red to green fluorescence. The emitted red and green fluorescence are detectable by flow cytometry. The ratio of red/green fluorescence represents the change of MMP in the cells. Figure 6a shows JC-1 fluorescence in both the FL-1 and FL-2 channels. The data presented here indicated that cells transfected with Hif-1aa exhibited high red fluorescence and weak green fluorescence compared with Hif-1ab, Myc-vector and control (*p* < 0.01, Fig. 6b), indicating that zebrafish Hif-1aa and Hif-1ab are functionally divergent in regulating MMP, and might therefore affected mitochondrial function and stability.

### Hif-1aa decreased cytosolic Ca^2+^ concentration

**Figure 7:**
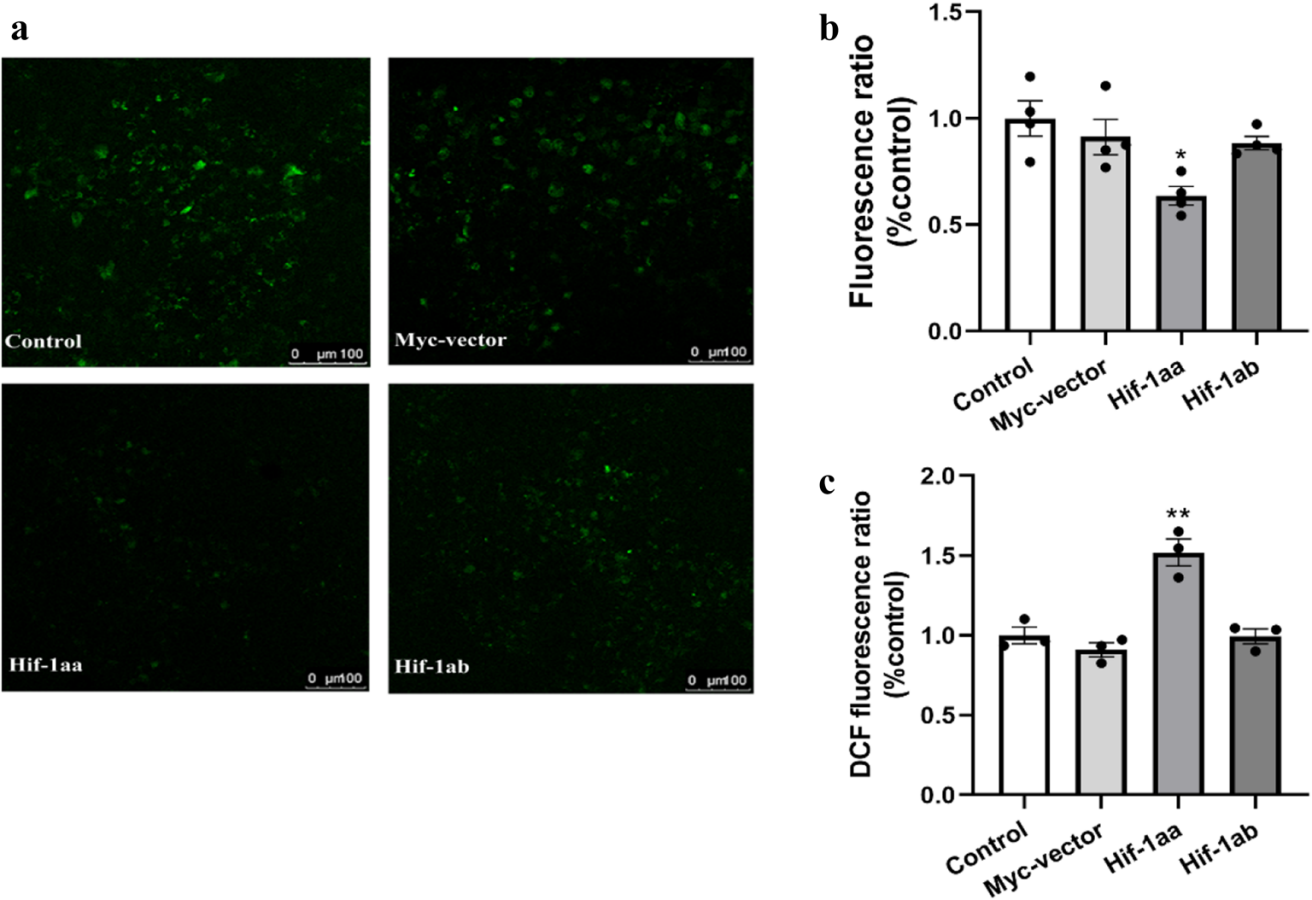
Disturbed Ca^2+^ homeostasis and ROS production regulated by zebrafish HIF-1aa and HIF-1ab. (**a**) Cells were stained with Fluo-4 AM to visualize intracellular Ca^2+^. Fluorescence intensity is correlated with the intracellular Ca^2+^ concentration. (**b**) Statistical analysis shows that HIF-1aa induced a decrease in the intracellular Ca^2+^ concentration. Data are given as mean ± SE (n = 4 per group). *p < 0.05. (**c**) ROS generation regulated by HIF-1aa. Cells were stained with 10 μM DCHF diacetate for 30 min. Oxidative metabolism was reflected in the conversion to dichlorofluorescein (DCF). Fluorescence was determined using a multifunctional microplate reader. Data are given as mean ± SE (n = 3 per group). **p < 0.01.

Ca^2+^ homeostasis is very important for maintaining mitochondrial membrane potential and oxidative phosphorylation^30^, a sharp increase of cytosolic free Ca^2+^ may activate degradative processes and organelle dysfunction, particularly that of mitochondria^31^. We therefore considered whether the two Hif-1a paralogs might regulate cytosolic free Ca^2+^. The result showed that compared with other treatment group and control group, the cytosolic Ca^2+^ concentration significantly decreased in cells transfected with hif-1aa (*p* < 0.05, Fig. 7a,b). We consider that Hif-1aa promotes Ca^2+^ flow into mitochondria accompanied by an increase of ΔΨm.

### Hif-1aa enhances intracellular ROS level

To investigate the roles of zebrafish Hif-1aa and Hif-1ab in regulating the generation of ROS, HEK 293 cells were cultured and treated with the non-fluorescent compound dichlorodihydrofluorescein diacetate, which is oxidized to highly fluorescent dichlorofluorescein in the presence of ROS. In this study, 293 cells were treated with 100 μM CoCl_2_ for 24 h, and result showed a significant increase in fluorescence intensity in 293 cells transfected with hif-1aa but not hif-1ab (*p* < 0.01, Fig. 7c), indicating Hif-1aa is the one paralog that can affect the production of intracellular ROS.

## Discussion

Hypoxia-inducible factors are recognized as the key transcriptional factors that regulate major changes in gene expression under insufficient oxygen supply. In mammals, some HIF target genes have been reported to be involved in angiogenesis, erythropoiesis and carbohydrate metabolism^32^. Since many animals such as fish live in an aquatic environment with varying oxygen tension, it is of great significance to study the oxygen-dependent gene expression of these animals. It has been demonstrated that the molecular response of fish to hypoxia is similar to the oxygen-dependent response of mammals including humans, they have the same hydroxylation sites in the oxygen dependent degradation domain (ODDD)^12,33^. The evolution of *hif* followed the two rounds of genome duplication experienced by the ancestor of vertebrates^34^. The teleost-specific WGD occurred in the teleost early evolution, only cyprinids retain each *A/B* paralogous pair in most euteleosts. The ancestral duplication is thought to provide genetic raw material for adaptation to variable oxygen tensions in this lineage. These duplicated hif-α paralogous pairs are found to possess the same principal domains as their mammalian counterparts, and importantly, they have evolved specialized roles in the response to hypoxia. Till now, study of the HIF gene pairs in very limited. Previous evolutionary analysis suggested that Hif-1aa and Hif-1ab were under different selection pressures, Hif-1aa evolved significantly faster than Hif-1ab, and the transcription of Hif-1aa decreased after hypoxic insult in adult zebrafish, implying that cyprinid Hif-1aa is a less sensitive oxygen regulator than other vertebrate HIF-1 proteins^11^. However, our work showed that both Hif-1aa and Hif-1ab maintained stabled on the translational level with an increased transcriptional activity under CoCl_2_ treatment.

We have shown that zebrafish Hif-1aa and Hif-1ab are functionally divergent in regulating mitochondrial function. Hif-1aa increases the activity of complex II (succinate dehydrogenase, SDH), which is responsible for electron generation during the conversion of succinate to fumarate and is also an important enzyme complex in both the electron transfer chain and the tricarboxylic acid cycle^35,36^. It’s been illustrated that inhibition of SDH results in succinate accumulation, which inhibits HIF-prolyl hydroxylases in the cytosol, leading to stabilization and activation of HIF-1α^37^. Therefore, the increase of the SDH activity by Hif-1aa would remove the inhibition of HIF-prolyl hydroxylases by succinate, forming into a negative feedback loop and keeping the expression of Hif-1aa and Hif-1ab in a proper physiological balance.

A previous study revealed a relationship between CII and CI, and considered that the destabilization of CI could be caused not only by CIII or CIV deficiency but also by activation of CII^38^. Once it was activated, the rate of ROS production was significantly increased by complex I, which induced a reverse electron flow from succinate to NAD^+^, and the reverse flow could also be enhanced by a high mitochondrial membrane potential (ΔΨm)^39,40^. In this study, Hif-1aa reduces the concentration of Ca^2+^ in cytoplasm and induces a high ΔΨm, we speculate that Hif-1aa promotes Ca^2+^ flow into mitochondria accompanied by an increase of ΔΨm, this process has been involved in previous study^41^. Importantly, Ca^2+^-induced permeability transition pore (PTP) opening also lead to the conformational change of complex I, which improved ROS (H_2_O_2_) production^42,43^. Taken together, we consider that Hif-1aa facilitates the ROS generation by multiple signaling pathways, the activation of CII, Ca^2+^ overload and a high ΔΨm. It has been demonstrated that the increased of ROS production can inhibit prolyl hydroxylase enzyme (PHD) activity, and prevent the degradation and enhance the stability of HIF-1^44^. However, excessive ROS may related to the cellular oxidative damage, such as mitochondrial dysfunction and mitophagy^45^. Hif-1aa improved ROS production and concomitant increase of mitochondrial membrane potential, suggesting that Hif-1aa maintains hyperpolarization of the mitochondrial membrane potential. Lambert et al. demonstrated that human HIF-1α inhibited apoptosis by promoting glycolysis and mitochondrial membrane potential hyperpolarization^46^. Therefore, this function likely existed in the ancestral gene of mammalian and teleost HIF-1α, and was lost in HIF-1αB after the duplication of cyprinid HIF-1α. In addition, the biological function of Ca^2+^ is multifaceted, some studies have demonstrated that the increase of Ca^2+^ in mitochondria causes the allosteric activation of TCA cycle enzymes including α-ketogluterate dehydrogenase and pyruvate dehydrogenase, as well as isocitrate dehydrogenase ^47,48^. Herein, we found Hif-1aa significantly improved the expression level of *citrate synthase*, which is also the key enzyme of TCA cycle. Therefore, we draw a conclusion that HIF-1αA plays a crucial role in TCA cycle under hypoxia, and a balance exists between the detrimental and the beneficial effects on mitochondria. Of the two paralogs, Hif-1ab is thought to be responsible for the hypoxic stress response because it is more stable at the beginning of a hypoxic insult^11^, but considering Hif-1aa regulates a series of signal pathways including ROS generation, Ca^2+^ overload, ΔΨm and complex II activity, we speculate that Hif-1aa can modulate the stability of itself and Hif-1ab in a multi-dimensional way.

Zebrafish Hif-1ab improved the activity of complex I, complex III and complex IV, and the expression level of relative genes. The OXPHOS system comprises five multisubunit complexes, which encoded by nuclear and mitochondrial genome except for complex II as it is encoded only by nuclear genome^49,50^. It is well known that the co-adaptation of nuclear and mitochondrial genes of the OXPHOS system is a crucial mechanism in adaptive evolution of organisms^51^. In this study, we found the correlation between nuclear and mitochondrial gene transcription in OXPHOS complexes I, III and IV, which suggests that the nuclear and mitochondrial genomic regions encoding this portion of the OXPHOS pathway are co-adapted in zebrafish Hif-1ab. However, nuclear and mitochondrial transcriptions are uncorrelated for the complex IV in zebrafish Hif-1aa, which enhanced the expression level of *COI*, but not *COX8A*. Previous studies have suggested that the decoupling of nuclear and mitochondrial transcription for OXPHOS complexes may be important for individual fitness^52,53^. Thus, herein we consider that the decoupling of nuclear and mitochondrial transcriptions for complex IV may be the adaptive response of Hif-1aa to hypoxia. Meanwhile, we demonstrated that Hif-1ab regulated the oxidative phosphorylation of mitochondria by enhancing the activities of complexes I, III and IV, thus directly modulating ATP generation.

Mitochondrial biogenesis is a complex process and can be induced by multiple signaling pathways^54–56^. Our study showed that Hif-1aa and Hif-1ab improved the transcription level of mtDNA control region, which contains the major regulatory elements for mtDNA replication and transcription^57,58^. Furthermore, we quantified mitochondrial mass by Mito-tracker Green, and the result showed that the relative fluorescence intensity was significantly increased in cells transfected with Hif-1aa and Hif-1ab. Altogether, we interpret these results to reveal that both preserved Hif-1a paralogs, Hif-1aa and Hif-1ab, in zebrafish significantly promote mitochondrial biogenesis, which restores mitochondrial mass and maintains mitochondrial homeostasis. This phenomenon likely explains the enhanced capacity of cyprinid fish to endure hypoxic conditions and their survival in freshwater environments through the evolutionary process.

In summary, we provide evidence that zebrafish Hif-1aa and Hif-1ab are functionally diverged in regulating mitochondrial function. The Hif-1aa plays a key role in TCA cycle by regulating the Ca^2+^ concentration, ROS production, mitochondrial membrane potential and the transcript level of *Citrate synthase*; while the Hif-1ab mainly promotes the oxidative phosphorylation and ATP generation. The two zebrafish Hif-1a paralogs regulate the metabolism and energy production (TCA cycle and OXPHOS), respectively. In addition, we also demonstrate that zebrafish Hif-1aa and Hif-1ab promote mitochondrial biogenesis in response to enhanced oxygen demand and maintain an optimal balance between the competing demands of energy and redox homeostasis responses to low oxygen levels.

## Methods

### Cell culture

Human embryonic kidney (HEK) 293 cells were maintained in Dulbecco’s modified Eagle’s medium (DMEM) (HyClone, Logan, UT, USA) containing 10% foetal bovine serum (FBS) (HyClone, Logan, UT, USA) and 1% penicillin–streptomycin (Invitrogen, Carlsbad, USA). The cells were cultured at 37 °C in a 5% CO_2_ incubator (Thermo). In some cases, HEK 293 cells were treated with CoCl_2_ (100 μM) (Sigma, St. Louis, USA) in 10% FBS medium and maintained for 24 h at 37 °C.

### Plasmid construction

The indicated Myc-tagged Hif-1a constructs, pTK-*Renilla* luciferase reporter, HRE-luciferase reporter and pCMV-Myc vector were obtained from Dr. Lihong Guan. Full, verified sequences of the *hif-1aa* and *hif-1ab* genes of zebrafish were subcloned into the pCMV-Myc vector (Clontech. Palo Alto, CA), and all recombinant plasmids were verified by DNA sequencing.

### Luciferase reporter assays

Eighteen hours before transfection, HEK 293 cells were seeded in 24-well plates with 10% FBS medium, then co-transfected with the indicated Myc-tagged HIF-1a constructs (or empty vector), HRE-luciferase reporter, and pTK-*Renilla* luciferase reporter by using Lipofectamine 2000 transfection reagent. After transfection, the culture medium was removed and replaced with OPTI-MEM medium (Vigorous) for 4 h, and then replaced with 10% FBS medium, which contained 100 μM CoCl_2_ and incubated for 24 h. The negative control was showed as the empty vector co-transfected with HRE-luciferase reporter and pTK-Renilla luciferase reporter without CoCl_2_ treatment. Luciferase activity was detected by using a Dual-Luciferase Reporter Assay System (Promega, Madison, WI, USA). And pTK-*Renilla* as the luciferase internal control reporter gene was used to normalize data, the relative luciferase activity was showed as the ratio of firefly luciferase to renilla luciferase.

### Total RNA isolation and quantitative polymerase chain reaction analysis (qPCR)

Total RNA was extracted using Trizol reagent (Invitrogen) according to the manufacturer’s protocol. The quality and quantity of RNA was determined by NanoDrop 2000 (Thermo Scientific, Waltham, MA, USA), agarose gel electrophoresis and a UV spectrophotometer (Thermo Scientific NanoDrop 2000). Total RNA was reverse transcribed into cDNA by using a cDNA Synthesis Kit (TaKaRa, Shiga, Japan). Ten genes were selected to detected the mitochondrial function (Table 1), consisting of mtDNA control region (D-Loop), *Citrate synthase*, four nuclear genes (Ubiquinone oxidoreductase subunit A1 (*NDUFA1*), Succinate dehydrogenase complex flavoprotein subunit A (*SDHA*), Ubiquinol-cytochrome c reductase core protein 1 (*UQCRC1*) and Cytochrome c oxidase subunit 8A (*COX8A*)) and three mitochondrial genes (Cytochrome c oxidase subunit I (*COI*), Cytochrome b (*Cytb*) and NADH-ubiquinone oxidoreductase chain 1 (*NADH1*)), and β-actin was used as an internal control. The mRNA level was assayed by the real-time qPCR method with a quantitative thermal cycler (MyiQTM 2 Two Color Quantitative PCR Detection System, Bio-Rad, Hercules, CA, USA). Calculations for relative expression were performed using the 2^-∆∆Ct^ method.

**Table 1:** Primer sequences for real-time PCR.

Genes	Primer sequences (forward/reverse)
MtDNA control region	5′ GGGAAGCAGATTTGGGTAC 3′/5′ GGTTGATGTGGATTGGGT 3'
NADH-Ubiquinone oxidoreductase subunit A1 (NDUFA1)	5′ TTCAAGGACCCAGAAGTAG 3′/5′ GACTCCAGTGATACCCAAA 3'
NADH-ubiquinone oxidoreductase chain 1(NADH 1)	5′ TAATGCTTACCGAACGAA 3′/5′ GGTGATGGTAGATGTGGC 3'
Succinate dehydrogenase complex flavoprotein subunit A (SDHA)	5′ CGAGAAGGAAGAGGCTGTG 3′/5′ GAATGCCGCCCATGTTAT 3'
Ubiquinol-cytochrome c reductase core protein 1 (UQCRC1)	5′ ACGCAGCCTCCTGACCTA 3′/5′ GAAGCGCAGCCAGAACAT 3'
Cytochrome b (Cytb)	5′ CCTGAAACATCGGCATTA 3′/5′ GGGTGGGACTGTCTACTG 3'
Cytochrome c oxidase subunit 8A (COX8A)	5′ GGTGTACTCCGTGCCATCA 3′/5′ CAGGAGGTAAGCCCAACG 3'
Cytochrome c oxidase subunit I (COI)	5′ CAGACCGCAACCTCAACA 3′/5′ CGAAGCCTGGTAGGATAA 3'
Citrate synthase	5′ CGCCTGTACCTCACCATC 3′/5′ TTGCCAACTTCCTTCTGC 3'
β-actin	5′ TGGGCATGGAGTCCTGTG 3′/5′ CTGCATCCTGTCGGCAAT 3'

### Mitochondrial mass analysis

Mitochondrial mass was determined by the fluorescent probe Mito-tracker Green (Beyotime, Jiangsu, China). Cells were incubated with 100 μM CoCl_2_ for 24 h after transfection, then incubated with the 100 nM Mito-tracker Green in pre-warmed DMEM culture media for 30 min at 37 °C in the dark. Fluorescence intensity was analyzed by Guava easyCyte 8HT flow cytometry (Merck Millipore, Bille-rica, MA, USA). In addition, cells were also incubated with a culture medium containing 100 nM Mito-tracker Green probe at 37 °C for 30 min, then were incubated with 5 μM Hoechst 33,342 (Sigma, St. Louis, USA) at 37 °C for 30 min before monitored under a Leica TCS SP confocal microscope (Leica Microsystems, Heidelberg, Germany).

### Assays for activities of Mitochondrial Complexes

Mitochondrial protein was collected using a cell mitochondrial kit from Beyotime in accordance with the manufacturer’s instructions. Then, the extracted mitochondrial proteins were used to detected the activities of the OXPHOS complex I, II, III and IV, which were performed by four kinase activity assay kits according to the manufacturer’s instructions (GeneMed Scientifics Inc., Arlington, MA, USA).

### Measurement of intracellular ROS

Intracellular ROS production was detected by the Reactive oxygen species Assay Kit (Nanjing Jian Cheng Bioengineering Institute, Nanjing, China). Cells were incubated in 10% FBS medium containing 100 μM CoCl_2_ for 24 h after transfection. Then cells were washed with PBS and treated with 100 nM DCFH-DA at 37 °C for 30 min in the dark and then analyzed by using Multifunctional microplate reader SpectraMax M5 (Molecular Devices, Sunnyvale, CA, USA).

### Mitochondrial membrane potential assay

Mitochondrial membrane potential was detected by the JC-1 (Beyotime, Jiangsu, China). Cells were maintained in 10% FBS medium containing 100 μM CoCl_2_ for 24 h after transfection, then incubated with 500 μl JC-1 staining solution at 37 °C in the dark for 20 min. Green (FL1 channel 530/30 nm band pass filter) and red (FL2 channel 585/42 nm band pass filter) fluorescences were determined by Guava easyCyte 8HT flow cytometry^59^. The cells sorting gates used were FL-2 versus FL-1 blotting and all data were analyzed by using Flow Jo 9.0 software (Tree Star, Ashland, OR, USA). Cells were also collected and incubated with Mito-tracker Red CMXRos probe (Molecular Probes, Eugene, OR) at 37 °C in the dark for 30 min monitored under a Leica TCS SP confocal microscope.

### Measurement of intracellular Ca^2+^ concentration

Ca^2+^ concentration measurement was performed by using the methods described previously^60^. Cells were exposed with 100 μM CoCl_2_ for 24 h before stained with 3 μM Fluo-4 AM (Sigma, St. Louis, USA) in HBSS (Gibco, Grand Island, NY, USA) at 37 °C for 30 min. And the Fluo 4-loaded cells were washed with Ca^2+^-free HBSS buffer to remove the excess extracellular Fluo-4 AM, then the Ca^2+^ signal was detected by a Leica TCS SP confocal microscope.

### Measurement of intracellular ATP content

The ATP content in cells were obtained using an enhanced ATP assay kit (Beyotime, Jiangsu, China) according to the manufacturer’s instructions. This method for ATP assay is based on a luciferase-luciferin reaction assay. In this study, cells were exposed with 100 μM CoCl_2_ for 24 h and split by the lysis reagent, then centrifugated at 12,000 g for 5 min to obtained supernatant. The protein concentration of samples was detected by BCA protein assay kit (Beyotime, Jiangsu, China) to avoid the error caused by the difference of protein content. Finally, the chemical fluorescence intensity of samples was measured by Multifunctional microplate reader SpectraMax M5 (Molecular Devices, Sunnyvale, CA, USA) and the concentration of ATP were calculated according to an ATP standard curve.

### Statistical analysis

All the experiments were presented at least three times with a minimum of three repeats. Statistical analysis was conducted with SPSS 16.0 software (SPSS Inc., Chicago, IL, USA). Results were performed as means ± standard error (SE). And the data were presented by one-way analysis of variance (ANOVA) and followed by Turkey’s multiple range tests. Statistical analysis was showed as dot plots with bar by Graphpad Prism 8.02 (Graphpad Software, San Diego, CA). The dot plots indicate the distribution of individual datapoints. In each analysis, a value of *P* less than 0.05 was considered to indicate statistical significance.

## Data Availability

All datasets generated for this study are included in the manuscript.
